# *In situ* formation of the first proteinogenically functionalized [TeW_6_O_24_O_2_(Glu)]^7–^ structure reveals unprecedented chemical and geometrical features of the Anderson-type cluster†
†Electronic supplementary information (ESI) available: Full experimental details and additional figures are provided. See DOI: 10.1039/c6cc07004c



**DOI:** 10.1039/c6cc07004c

**Published:** 2016-09-06

**Authors:** Christian Molitor, Aleksandar Bijelic, Annette Rompel

**Affiliations:** a Universität Wien , Fakultät für Chemie , Institut für Biophysikalische Chemie , Althanstraße 14 , 1090 Wien , Austria . Email: annette.rompel@univie.ac.at ; http://www.bpc.univie.ac.at

## Abstract

The chemistry of polyoxometalates (POMs) in a protein environment is an almost unexplored but highly relevant research field as important biological and pharmacological attributes of certain POMs are based on their interactions with proteins. We report on the A-type Anderson–Evans polyoxotungstate, [TeW_6_O_24_]^6–^ (TEW), mediated crystallization of *Coreopsis grandiflora* aurone synthase (*cg*AUS1) using ∼0.24 mM protein and 1.0 mM TEW. The 1.78 Å crystal structure reveals the covalent binding of TEW to the protein under the formation of an unprecedented polyoxotungstate cluster, [TeW_6_O_24_O_2_(Glu)]^7–^ (GluTEW). The polyoxotungstate–protein complex exhibits the first covalent bond between a protein and the A-type Anderson–Evans cluster, an archetype where up to now no hybrid structures exist. The polyoxotungstate is modified at two of its six addenda tungsten atoms, which covalently bind to the carboxylic oxygen atoms of glutamic acid (Glu157), leading to W–O distances of ∼2.35 Å. This ligand substitution reaction is accompanied by a reduction of the coordination number of two μ_3_ polyoxotungstate oxygen atoms. This is so far unique since all known hybridizations of the Anderson–Evans POM with organic units have been obtained *via* the functionalization of the B-type Anderson–Evans structure through its bridging oxygen atoms. The structure reported here proves the reactivity of this POM archetype's addenda atoms as it has been administered into the protein solution as a pre-assembled cluster. Moreover, the novel cluster [TeW_6_O_24_O_2_(Glu)]^7–^ displays the great versatility of the Anderson–Evans POM class.

## Abstract 2

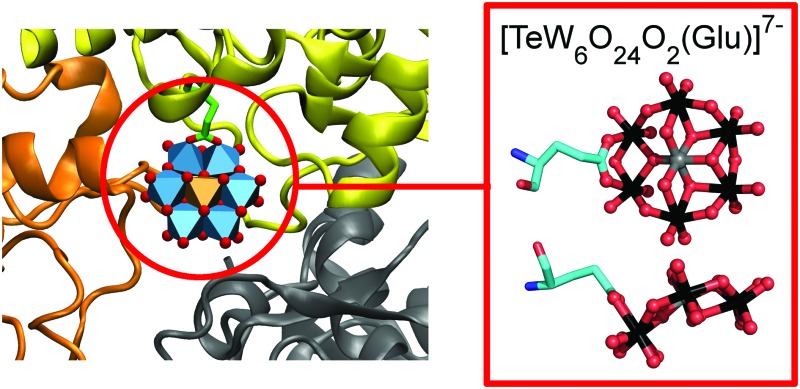
Protein crystallographic investigations using the well-known Anderson-type polyoxometalate as crystallization additive led to the *in situ* formation of the unprecedented and proteinogenically functionalized [TeW_6_O_24_O_2_(Glu)]^7–^ cluster.

Polyoxometalates (POMs) represent a diverse family of anionic metal oxide clusters with a broad variety of structures and outstanding properties, thus, having a wide spectrum of applications.^1^ Thereby, POM–protein interactions become more and more the focus of this research field since most of the reported POM-related biological and/or pharmacological attributes are based on their interaction with proteins.^2^ Biomolecular applications exploiting POM–protein interactions are, among others, the usage of POMs as crystallization agents, artificial proteases and enzyme inhibitors.^3–6^ In protein crystallography POMs have primarily been used for derivatization reasons in order to solve the phase problem.^2*c*^ The most prominent usage has been the derivatization of the ribosomal subunits by various POM anions (*e.g.* [PW_11_O_39_]^7–^, [PW_12_O_40_]^3–^, and [P_2_W_18_O_62_]^6–^).^3^ However, it must be noted that Steitz and co-workers obtained the best diffracting data set when applying an osmium derivative.^3*a*^ The hydrolytic activity of POMs towards the peptide bonds in biomolecules and other model oligopeptides has been studied revealing selective hydrolysis by certain Zr^4+^ or Ce^4+^ containing POMs, making them artificial metalloproteases.^4^ POMs, especially vanadates, have also been explored to act as powerful inhibitors of phosphatases and ATPases, which is of great biological interest considering the importance of phosphorylation reactions in signal transduction cascades.^6^

One of the most prominent POM archetypes is the Anderson–Evans structure,^7,8^ which is composed of six edge-sharing MO_6_ (M = Mo or W) octahedra surrounding an octahedral edge-sharing heteroatom leading to an approximate *D*_3d_ geometry. The Anderson–Evans structure possesses six triple-bridged (μ_3_-O), six double-bridged (μ_2_-O) and twelve terminal (O_t_) oxygen atoms. There exist two kinds of Anderson POMs, namely the A- and B-types. The A-type is non-protonated and its central heteroatom exhibits the highest oxidation state, whereas the B-type contains up to six protons on the six μ_3_-O atoms and its heteroatom is thus found in lower oxidation states.

Applications for the pure inorganic Anderson–Evans POMs are rare; however, the application field of the A-type Anderson-polyoxotungstate, [TeW_6_O_24_]^6–^ (TEW),^9^ has recently been expanded to its successful use as an additive in protein crystallization.^10–12^ TEW demonstrated superiority not only over other POM archetypes but also over commonly used crystallization additives due to its good water solubility, pH-stability, disk-shape structure and relatively high negative charge. So far it has been observed that TEW does not change the protein's structure nor does it affect the protein's integrity, but provides a useful anomalous signal for phasing due to its six heavy tungsten atoms.

To understand better the interaction between TEW and a protein, we tried to obtain protein crystals in the presence of TEW. This was achieved for the latent form of the metalloprotein aurone synthase from *Coreopsis grandiflora* (*cg*AUS1)^10*c*^ which crystallized into different crystal forms by solely exchanging the additive magnesium chloride (100 mM) for TEW (1 mM).^10a,b^ In the absence of TEW two crystal forms were obtained: Cryst1, space group *P*12_1_1, 4 monomers per asymmetric unit (ASU) (PDB code: ; 4Z11), and Cryst2, space group *P*1, 8 monomers per ASU (PDB code: ; 4Z14). Both crystal forms diffracted X-rays only weakly leading to moderate resolutions. However, the crystal form obtained in the presence of TEW (CrystTEW, space group *P*12_1_1, 2 monomers per ASU, PDB code: ; 4Z13) was of significant higher quality and diffracted to 1.93 Å representing an improvement of up to 1.0 Å (at 〈*I*/*σI*〉 = 2) in comparison to the “TEW-less” crystal forms and the structure has been refined to 1.78 Å.^10*a*^

The asymmetric unit of the *cg*AUS1–TEW structure contains two *cg*AUS1 monomers and three TEW molecules, whereby two of the TEW anions are located within positively charged interfaces of three adjacent *cg*AUS1 monomers (Fig. 1) and the third one is solely bound to one *cg*AUS1 monomer (chain A) and displays a blurred electron density. One of the two TEW anions (TEW in Fig. 1) positioned at a positively charged patch is electrostatically interacting with five very flexible lysine residues (originating from three different protein monomers) (Fig. S1a and S2b, ESI†) leading to a not well-defined electron density for the lysine side chains.

**Fig. 1:**
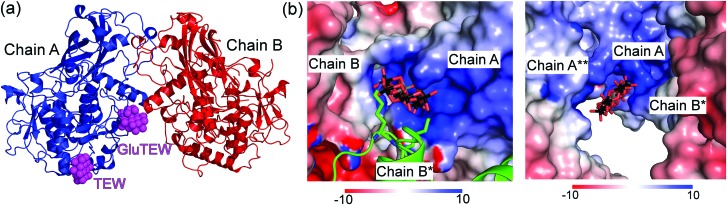
Asymmetric unit of CrystTEW and electrostatic potential of the binding pockets of GluTEW and TEW. (a) Representation of the asymmetric unit of CrystTEW. The interfacing GluTEW and TEW are presented as magenta spheres. The third chain B* was omitted for clarity. (b) GluTEW and TEW bind to a positively charged cleft and a patch, respectively. The molecular surface of the monomers is colored by the electrostatic potential on the solvent accessible surface (scale in kT e^–1^). Left: GluTEW binds within the interface of three *cg*AUS1 monomers. The symmetry related chain B* is visualized in cartoon/stick representation. Right: Binding site of TEW.

Remarkably enough, the second TEW (GluTEW in Fig. 1), which is located within a more narrow interface, displays a well-defined electron density (Fig. 2) revealing that two tungsten addenda atoms are covalently bound to the carboxylic oxygen atoms (Oε1 and Oε2) of residue Glu157 (Fig. 2a), which leads to the formation of a new TEW-derived cluster with the formula [TeW_6_O_24_O_2_(Glu)]^7–^ (GluTEW). The W–O(Glu) distances in GluTEW (∼2.35 Å) are slightly longer than the reported Mo–O(Glu) (2.11 Å) distances observed in the covalent binding between octamolybdate and the molybdenum storage protein, where only one oxygen atom (Oε1) of Glu129 is covalently bound to one Mo atom of the octamolybdate.^13^ This difference can be explained by the fact that in the case of GluTEW both carboxylic oxygen atoms bind to one tungsten atom each. Thus, the electrons participating in the covalent bond are delocalized over the W–O–C(Glu)–O–W bonds. As GluTEW is located deep inside a positively charged cleft of chain A (Fig. 1b and Fig. S2, ESI†), it can be assumed that the covalent binding of Glu157 is sterically enforced by the strong interactions of the surrounding amino acid side chains.

**Fig. 2:**
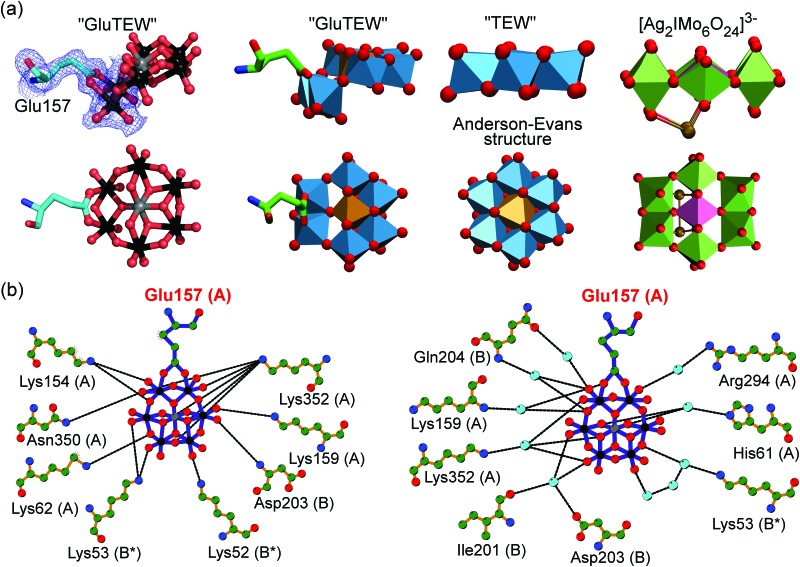
Covalent binding of GluTEW resulting in a novel polyoxotungstate. (a) Left: The carboxylic oxygen atoms of Glu157 bind covalently to two tungsten atoms of hexatungstotellurate(vi) (W–O distance ∼2.35 Å), while a reduction of the coordination number of two tridentate oxygen atoms occurs. A partially 2Fo–Fc electron density map (blue mesh) contoured at 1.0*σ* is shown. Right: Structure comparison of TEW, GluTEW and the polyoxomolybdate [Ag_2_IMo_6_O_24_]^3–^.^14^ (b) Analysis of GluTEW–protein interactions using LigPlot^+^.^15^ Left: Direct TEW–*cg*AUS1 hydrogen interactions. Right: Water-mediated TEW–*cg*AUS1 hydrogen interactions. Color code: (a) ball-and-stick presentation: carbon, light-cyan; nitrogen, blue; oxygen, red; tungsten, black; tellurium, grey. Polyhedra presentation: carbon, green; nitrogen, blue; oxygen, red; tungsten, marine-blue; tellurium, light-brown; silver, gold; molybdenum, forest-green; iodine, purple. (b) Small sphere presentation: carbon, green; nitrogen, blue; oxygen, red; tungsten, black; tellurium, grey; water molecules, light-cyan.

The covalent binding of Glu157 to the tungsten atoms can be best described as a ligand substitution reaction accompanied by the breakage of two tridentate W–μ_3_-O bonds resulting in an *in situ* formation of the novel cluster [TeW_6_O_24_O_2_(Glu)]^7–^ (Fig. 2a). This alteration of the binding mode of some of the TEW's oxygen atoms leads to a bent structure (Fig. 2a) and demonstrates the flexibility of the “planar” Anderson type POM in the presence of proteins. Although GluTEW is structurally closely related to the Anderson–Evans structure, it cannot be unambiguously classified in this POM archetype as it does not possess the characteristic edge-sharing hexameric addenda ring anymore. Instead, GluTEW contains a mixed edge/corner sharing addenda hexamer resulting in a structural mixture between a classical Anderson–Evans structure and the polyoxomolybdate [Ag_2_IMo_6_O_24_]^3–^ ^14^ (Fig. 2a). Therefore, GluTEW is best described as a two-corner four-edge sharing Anderson–Evans derived cluster.

So far, covalent binding between a POM and a protein was only observed when the POM was *in situ* assembled in the presence of the protein, for example, the covalent interactions between octamolybdate and NTPDase1 after oxygen ligand exchange by a serine and histidine side chain.^16^ Other examples are the crystal structures of the already above-mentioned molybdenum storage protein, where a self-assembled octamolybdate is covalently bound to a histidine and a glutamic acid.^16^ However, to the best of our knowledge, the formation of a covalent bond between a protein and a POM, which was administered as an intact cluster to the protein solution, has not been reported before. GluTEW represents the first modified A-type structure, which, together with the very recently reported first tris-functionalized (tris = tris(hydroxymethyl)methane (RC(CH_2_OH)_3_)) B-type structure, expands the great chemical versatility of this POM-archetype.^17^ Our findings demonstrate that not only the protonated μ_3_-O atoms of the B-type Anderson–Evans polyoxotungstate are accessible for functionalization but also the addenda atoms. Therefore, it can be assumed that the tungsten atoms of polyoxotungstates are generally accessible and thus modifiable by organic units (here the protein) with one of the most common functionalities, the carboxyl group (here provided by Glu157).

Besides the covalent binding, the terminal and bridging oxygen atoms from GluTEW interact with in total twelve protein residues, originating from three different *cg*AUS1 monomers, either directly *via* hydrogen bonds (Fig. 2b, left) or through a network of water bridges (Fig. 2b, right). The GluTEW anion is predominantly bound by side chains of positively charged residues. However, nitrogen and oxygen atoms of the protein backbone are also participating in the GluTEW–protein interactions. Notably, even an acidic residue (Asp203, chain B) is interacting with the terminal oxygen atoms of GluTEW through a water bridge (Fig. 2b, right).

The location and interactions of GluTEW within the protein structure not only reveal the beneficial role of the POM to mediate crystal contacts by cross-linking different protein monomers but also the highly advantageous scaffold of the TEW anion. Due to its disk shape, TEW enables multifaceted possibilities of its incorporation within the protein–protein interfaces: while the non-covalently bound TEW (TEW in Fig. 1) is positioned in a relative huge void of three protein molecules, GluTEW is immersed deeply inside a small positive cleft revealing that the disk shaped POM is not a rigid construct but is sterically flexible (Fig. 2 and Fig. S1, ESI†). Highly important for TEW use in protein crystallization is the fact that GluTEW formation does not alter the overall structure of the protein but stabilizes otherwise flexible loop regions (see Fig. S3, ESI†).

In summary, we have shown the formation of a covalent bond between a protein and a polyoxotungstate, which led to a novel modified A-type Anderson-like cluster (GluTEW) possessing a bended structure and thus demonstrating a special kind of flexibility with respect to both geometric and functional properties. So far (organic–inorganic) hybrid structures of A-type Anderson archetypes do not exist in the literature. Thus, our results evidence, for the first time, that a functionalization of A-type Anderson–Evans POMs by the decoration of its addenda atoms (here: W) with a carboxylic group is possible. The here reported functionalization of the A-type Anderson POM strongly depends on the sterical and electrostatical environment. Therefore, reaction conditions and the exact circumstances like chemical/electrostatic environment under which the addenda atoms are accessible in a scalable synthesis have to be developed.

This research was funded by the Austrian Science Fund (FWF): P25217 and P27534. We thank the beamline scientists Elspeth Gordon (ESRF ID23-1, mx1450), Anja Burkhardt (DESY P11, I-20120633 EC) and Alice Douangamath (Diamond Light Source I04-1, MX8476) for their generous support during the allocated beam times. The authors wish to thank Amir Blazevic, MSc, Dr Nadiia I. Gumerova, Ioannis Kampatsikas, MSc, Dr Stephan G. Mauracher, Dipl.-Ing. Matthias Pretzler and Referees 1–4 of this manuscript for valuable discussions concerning this work.
