# Supplementary material for: In situ formation of the first proteinogenically functionalized [TeW6O24O2(Glu)]7– structure reveals unprecedented chemical and geometrical features of the Anderson-type cluster
Source: Chem Commun (Camb). 2016 Sep 6;52(83):12286–9. doi: 10.1039/c6cc07004c (PMC5066560; doi:10.1039/c6cc07004c)
Supplement: Supplementary file 3 [file CC-052-C6CC07004C-s003.pdf]

Electronic Supplementary Material (ESI) for Chemical Communications.
This journal is © The Royal Society of Chemistry 2016

Electronic Supplementary Information

In situ formation of the first proteinogenically functionalized $[TeW_6O_{24}O_2(Glu)]^{7-}$ structure reveals unprecedented chemical and geometrical features of the Anderson-type cluster

Christian Molitor, Aleksandar Bijelic and Annette Rompel*

Universität Wien, Fakultät für Chemie, Institut für Biophysikalische Chemie, Althanstraße 14, 1090 Wien, Austria.

* annette.rompel@univie.ac.at; http://www.bpc.univie.ac.at

Content

1. Methods …… 3

1.1. Purification, crystallization, data collection and structure determination of *cg*AUS1……………… 3

1.2. Structure analysis and graphical representation ……………………………………………………………… 3

2. Additional Figures ……… 4

3. References …… 7

1. Methods

1.1. Purification, crystallization, data collection and structure determination of *cg*AUS1

The protein purification, protein crystallization, data collection and the data processing has been described previously.[1,2,3] The structures were solved and refined using Phenix[4] as described previously.[5] Restraints for the polyoxometalate hexatungstotellurate(VI) (TEW) were obtained with PHENIX.reel and PHENIX.elbow as reported previously[6,7,8] using the crystal structure of TEW.[9] Restraints for GluTEW were obtained analogously after placing the atoms into the electron density. The quality of the models was validated by MOLPROBITY.[10] The models were refined using PHENIX.refine. Weak data (mean $I/\sigma I < 2$) were included in the final refinement, in the case that they improved the models and their stereochemistry.[11] The final refinement statistics have been presented previously[5] and the models have been deposited in the PDB (http://www.rcsb.org) as entries 4Z11 and 4Z13 corresponding to data sets 4 and 7, respectively in Molitor et al..[3]

1.2. Structure analysis and graphical representation

Calculations for the electrostatic surface potential of pro-AUS1 were performed using the PDB2PQR[12] webserver and the APBS[13] webserver. Protein-POM interactions were analyzed and visualized with LigPlot$^+$.[14] Molecular graphics images were generated with PyMOL (http://www.pymol.org/) and VMD.[15]

2. Additional Figures

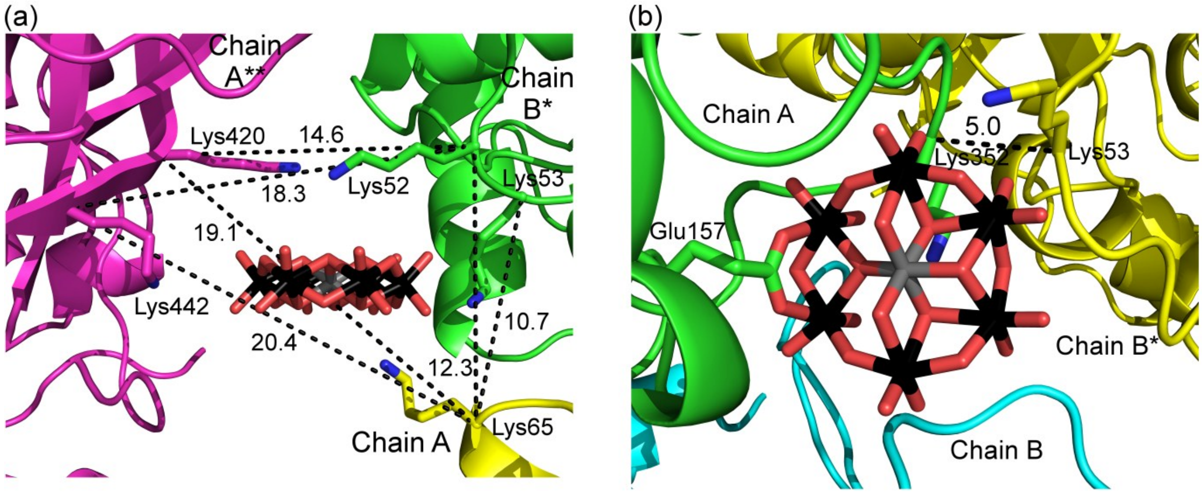

Fig. S1. Comparison of the binding sites of TEW and GluTEW. (**a**) TEW interacts with 5 lysine residues originating from three different pro-AUS1 monomers. The distance of their Cα atoms range between 10 and 20 Å (shortest distance: 10.7 Å). (**b**) Due to its binding deep inside a cleft, the shortest intermolecular distance between Cα atoms of GluTEW binding residues is as short as 5.0 Å.

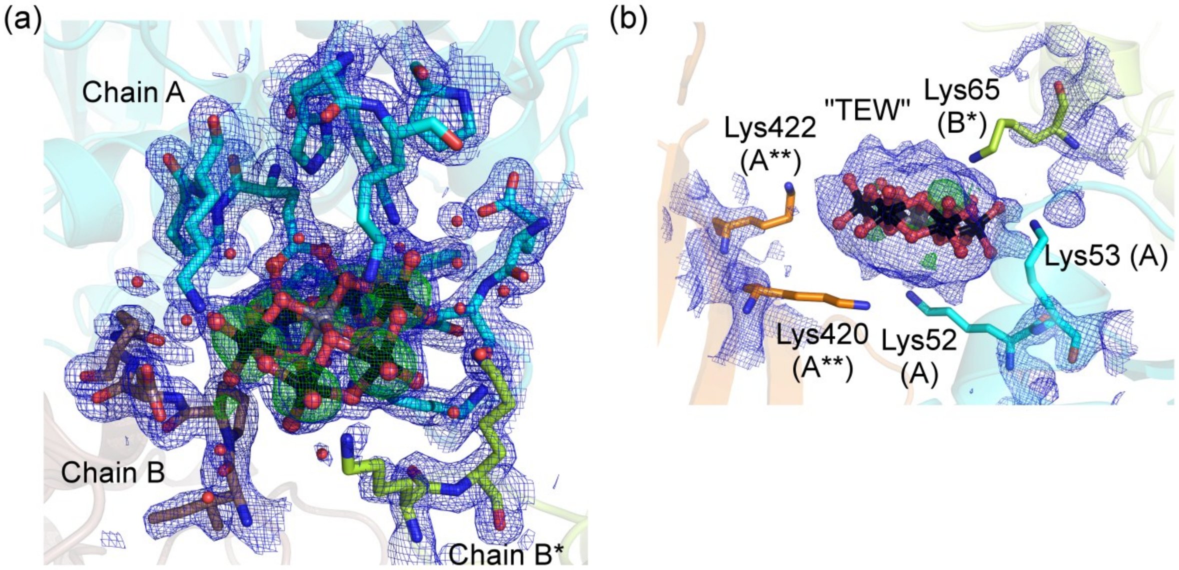

Fig. S2. Comparison of the electron densities of the GluTEW and TEW binding sites. The 2Fo-Fc electron density maps (blue mesh) are contoured at 1.0 σ and the anomalous Fourier difference maps (green mesh) are contoured at 3.0 σ. (**a**) The binding site of GluTEW possesses a well-defined electron density. (**b**) The binding site of TEW displays a not very well defined electron density.

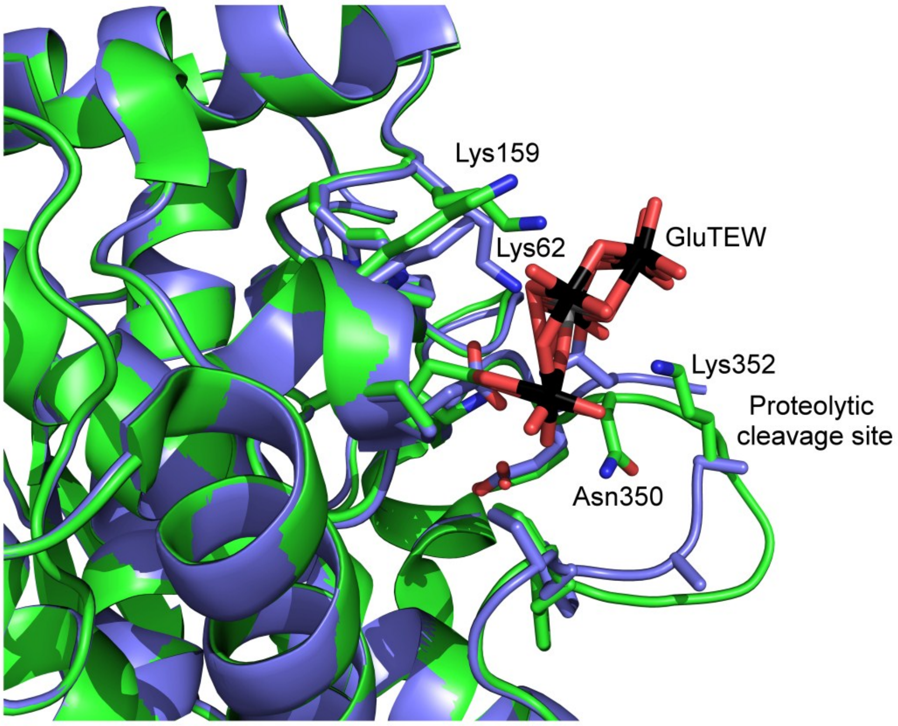

Fig. S3. Superimposition of CrystTEW (chain A) with Cryst1 (chain A) around the GluTEW binding site. The structure of CrystTEW is colored green and the structure of Cryst1 is colored blue. Due to the binding of GluTEW, the flexible loop region carrying one of three proteolytic cleavage sites of *cg*AUS1[5] is stabilized. Beside minimal conformational changes of the GluTEW binding side chains, no structural changes were observed upon the GluTEW binding.

3. References

1. C. Kaintz, C. Molitor, J. Thill, I. Kampatsikas, C. Michael, H. Halbwirth and A. Rompel, *FEBS Lett.* 2014, **588**, 3417-3426.

2. C. Molitor, S. G. Mauracher, S. Pargan, R. L. Mayer, H. Halbwirth and A. Rompel, *Planta* 2015, **242**, 519-537.

3. C. Molitor, S. G. Mauracher and A. Rompel, *Acta Crystallogr., Sect. F: Struct. Biol. Commun.*, 2015, **71**, 746–751.

4. P. D. Adams, P. V. Afonine, G. Bunkóczi, V. B. Chen, I. W. Davis, N. Echols, J. J. Headd, L.-W. Hung, G. J. Kapral, R. W. Grosse-Kunstleve, A. J. McCoy, N. W. Moriarty, R. Oeffner, R. J. Read, D. C. Richardson, J. S. Richardson, T. C. Terwilliger and P. H. Zwart, *Acta Crystallogr., Sect. D: Biol. Crystallogr.,* 2010, **66**, 213-221.

5. C. Molitor, S. G. Mauracher and A. Rompel, *Proc. Natl. Acad. Sci. U.S.A.*, 2016, **113**, E1806–E1815.

6. S. G. Mauracher, C. Molitor, R. Al-Oweini, U. Kortz and A. Rompel, *Acta Crystallogr., Sect. F: Struct. Biol. Commun.*, 2014, **70**, 263–266.

7. S. G. Mauracher, C. Molitor, R. Al-Oweini, U. Kortz and A. Rompel, *Acta Crystallogr., Sect. D: Biol. Crystallogr.*, 2014, **70**, 2301–2315.

8. A. Bijelic, C. Molitor, S. G. Mauracher, R. Al-Oweini, U. Kortz and A. Rompel, *ChemBioChem*, 2015, **16**, 233–241.

9. K. J. Schmidt, G. J. Schrobilgen and J. F. Sawyer, *Acta Crystallogr., Sect. C: Cryst. Struct. Commun*., 1986, **42**, 1115–1118.

10. V. B. Chen, W. B. Arendall III, J. J. Headd, D. A. Keedy, R. M. Immormino, G. J. Kapral, L. W. Murray, J. J. Richardson and D. C. Richardson, *Acta Crystallogr., Sect. D: Biol. Crystallogr.,* 2010, **66**, 12-21.

11. P. A. Karplus and K. Diederichs, *Science* 2012, **336**, 1030-1033.

12. T. J. Dolinsky, J. E. Nielsen, J. A. McCammon and N. A. Baker, *Nucleic Acids Res.* 2004, **32***,* W665-667.

13. N. A. Baker, D. Sept, S. Joseph, M. J. Holst and J. A. McCammon, *Proc. Natl. Acad. Sci. U.S.A.,* 2001, **98**, 10037-10041.

14. R. A. Laskowski and M. B. Swindells, *J. Chem. Inf. Model,* 2011, **51**, 2778-2786.

15. W. Humphrey, A. Dalke and K. Schulten, *J. Mol. Graph,* 1996, **14**, 33-38.
